# Genetic Networks Required to Coordinate Chromosome Replication by DNA Polymerases α, δ, and ε in *Saccharomyces cerevisiae*

**DOI:** 10.1534/g3.115.021493

**Published:** 2015-08-21

**Authors:** Marion Dubarry, Conor Lawless, A. Peter Banks, Simon Cockell, David Lydall

**Affiliations:** * Institute for Cell and Molecular Biosciences, Faculty of Medical Sciences, Newcastle University, Newcastle upon Tyne, NE2 4HH, United Kingdom; † High Throughput Screening Facility, Newcastle Biomedicine, Newcastle University, Newcastle upon Tyne, NE2 4HH, United Kingdom; ‡ Bioinformatics Support Unit, Faculty of Medical Sciences, Newcastle University, Newcastle upon Tyne, NE2 4HH, United Kingdom

**Keywords:** DNA replication, DNA polymerase, Profilyzer, DIXY, quantitative fitness analyses (QFA), *Saccharomyces cerevisiae*

## Abstract

Three major DNA polymerases replicate the linear eukaryotic chromosomes. DNA polymerase α-primase (Pol α) and DNA polymerase δ (Pol δ) replicate the lagging-strand and Pol α and DNA polymerase ε (Pol ε) the leading-strand. To identify factors affecting coordination of DNA replication, we have performed genome-wide quantitative fitness analyses of budding yeast cells containing defective polymerases. We combined temperature-sensitive mutations affecting the three replicative polymerases, Pol α, Pol δ, and Pol ε with genome-wide collections of null and reduced function mutations. We identify large numbers of genetic interactions that inform about the roles that specific genes play to help Pol α, Pol δ, and Pol ε function. Surprisingly, the overlap between the genetic networks affecting the three DNA polymerases does not represent the majority of the genetic interactions identified. Instead our data support a model for division of labor between the different DNA polymerases during DNA replication. For example, our genetic interaction data are consistent with biochemical data showing that Pol ε is more important to the Pre-Loading complex than either Pol α or Pol δ. We also observed distinct patterns of genetic interactions between leading- and lagging-strand DNA polymerases, with particular genes being important for coupling proliferating cell nuclear antigen loading/unloading (Ctf18, Elg1) with nucleosome assembly (chromatin assembly factor 1, histone regulatory HIR complex). Overall our data reveal specialized genetic networks that affect different aspects of leading- and lagging-strand DNA replication. To help others to engage with these data we have generated two novel, interactive visualization tools, DIXY and Profilyzer.

DNA replication requires the coordinated action of numerous proteins and nucleic acids to ensure accurate genome duplication. At the core of the replicative machinery in eukaryotes are three B-family DNA polymerases. The three polymerases, Pol α, Pol δ, and Pol ε, work in concert with many other accessory proteins. For example, the six-subunit complex, the origin recognition complex, binds at replication origins throughout the cell cycle and recruits replication proteins to initiate replication. The origin recognition complex helps recruit the hexameric helicase MCM in G1, along with Cdc45 and GINS (CMG for Cdc45-MCM-GINS) in S-phase to initiate replication and unwind DNA at the front of the replication fork. The five-polypeptide complex, replication factor C (RFC), the sliding clamp loader, loads the trimeric sliding clamp, proliferating cell nuclear antigen (PCNA), onto DNA. PCNA helps tether polymerases to DNA to improve processivity during strand elongation (Waga and Stillman 1998).

DNA polymerase α-primase (Pol α) initiates replication of both leading and lagging strands (Georgescu *et al.* 2015). To perform this task, Pol α synthesizes a short RNA primer that is extended first by Pol α DNA polymerase activity before other DNA polymerases take over. The most widely accepted current view is that DNA polymerase δ (Pol δ) completes lagging-strand synthesis, whereas DNA polymerase ε (Pol ε) completes leading-strand synthesis (Garg and Burgers 2005). An alternative model is that Pol δ is the major polymerase for both leading and lagging strands and Pol ε is a repair polymerase (Johnson *et al.* 2015; Waga and Stillman 1994). Pol α has limited processivity and lacks intrinsic 3′ exonuclease activity for proofreading functions (Kunkel *et al.* 1989). The current evidence indicates that Pol α synthesizes about 1.5% of the genome (Reijns *et al.* 2015). In budding yeast, all four subunits of Pol α, Pri1, Pri2, Pol1, and Pol12, are essential for cell viability.

Pol δ principally completes synthesis of the lagging strand. In budding yeast Pol δ consists of three subunits: Pol3, Pol31, and Pol32. Pol3 is the catalytic subunit, and it has an associated 3′ exonuclease activity that can remove misincorporated nucleotides. Pol31, like Pol3, is essential in budding yeast. Pol32 is a nonessential subunit of Pol δ that help stabilize the other two subunits. The subunits of Pol δ play additional roles since, for example, Pol31 and Pol32 also physically interact with Rev3 and by this criterion are subunits of the error-prone DNA polymerase zeta (Pol ζ), which catalyses synthesis opposite DNA lesions (Kochenova *et al.* 2015).

Pol ε principally completes synthesis of the leading-strand. Pol ε is a four-subunit complex consisting of Pol2, Dpb2, Dpb3, and Dpb4. Pol2, the catalytic subunit, and Dpb2 are essential proteins in yeast, whereas Dpb3 and Dpb4 can be deleted. Pol2 DNA polymerase and 3′ exonuclease activities are located in the N-terminal domain while its C-terminal domain carries conserved zinc-finger motifs (Supporting Information, Figure S1). Interestingly, the C-terminus of Pol2 is essential for yeast viability, whereas the N-terminal polymerase domain of Pol2 is not (Dua *et al.* 1999; Feng and D’Urso 2001; Kesti *et al.* 1999). The current model to explain the fact that yeast cells can survive in the absence of the leading-strand DNA polymerase function is that the lagging-strand DNA polymerase Pol δ can, if necessary, replace Pol ε polymerase to replicate the leading strand (Daigaku *et al.* 2015). Additionally, Pol δ can replace Pol ε to proofread errors created by defective Pol ε and can complete DNA synthesis on the leading strand (Flood *et al.* 2015). It is thought that the most important (essential) function of Pol ε is to help initiate DNA replication as part of the preloading complex (pre-LC), working in concert with the GINS, Sld2, and Dpb11 proteins (Sengupta *et al.* 2013). Dpb3 and Dpb4, the nonessential subunits affect Pol ε binding to DNA and its processivity (Aksenova *et al.* 2010; Araki *et al.* 1991; Tsubota *et al.* 2003).

In eukaryotes, three multi-subunit polymerases interact to complete DNA replication, whereas in bacteria a single DNA polymerase is sufficient. The reasons for this difference are unclear but may reflect that fact that in eukaryotes, unlike prokaryotes, numerous origins are used to replicate each chromosome and eukaryotic chromosomes are linear, bringing an additional “end replication problem.” At each eukaryotic replication origin, a pair of replisomes travels in opposite directions from the origin (Masai *et al.* 2010) and will encounter replisomes coming in the opposite direction as well as other obstacles such as nucleosomes, DNA barrier elements, transcription factors, transcribing RNA polymerases, and damaged DNA, as well as chromosome ends (Tourriere and Pasero 2007). Accordingly, a large number of mechanisms have evolved to help the DNA replication machinery to deal with such challenges (Yeeles *et al.* 2013). Interestingly, it seems that distinct mechanisms have evolved to regulate the stability of leading- and lagging-strand machinery (Kurth and O’Donnell 2013).

Genetic suppressor and enhancer screens are a powerful way to explore how complex biochemical processes work *in vivo* and in particular how processes are controlled and interact with other cellular components or pathways. We have previously performed suppressor and enhancer analysis of telomere defective strains to identify pathways that are important for maintaining functional chromosome ends in budding yeast (Addinall *et al.* 2008, 2011). Here, we applied quantitative fitness analysis (QFA) to identify factors that affect fitness of strains defective in DNA replication. We identify numerous genes that affect general as well as specific aspects of eukaryotic DNA replication. To help others engage with this resource we also provide web-based tools that permit interaction with these and similar data sets.

## Materials and Methods

### Creation of the double-mutant libraries by synthetic genetic array (SGA)

Genotypes of the strains used in this study are described in Table S1. The thermosensitive (ts) alleles have been resequenced and mutations are shown in Figure S1. The SGA technique (Tong and Boone 2006; Tong *et al.* 2001) was used to combine the yeast genome knock-out (*yfgΔ*) and DAmP [decreased abundance by mRNA perturbation, which affects the function of essential genes (Schuldiner *et al.* 2005), (*yfg-d*)] collections with the *lyp1Δ*, *pol1-4*, *pol2-12*, *cdc2-2*, and *cdc13-1* mutations. The recessive, ts-mutated alleles from (Li *et al.* 2011b) were flanked by the selectable *LEU2* and HphMX markers in strains bearing *can1*::*STE2pr-SpHis5* and *lyp1*::NatMX. Diploids were selected on rich media containing geneticin, hygromycin B, and nourseothricin. Diploids were sporulated on solid medium, and meiotic haploid *MAT***a** double-mutant progeny were isolated on SC medium containing canavanine, thialysine, geneticin, hygromycin B, and nourseothricin while lacking leucine, lysine, histidine, and arginine. Selection was based on haploid selection markers (*can1*::*STE2pr-SpHis5*), double-mutant selection markers (KanMX, HphMX, NatMX, and *LEU2*), and thialysine (*lyp1*::NatMX). Solid agar to solid agar pinning was performed on a Biomatrix BM3-SC robot (S&P Robotics Inc., Toronto, Canada) at 20° as previously described in Addinall *et al.* (2011).

### Spotting yeast cultures for QFA

Double mutants obtained from SGA were inoculated into liquid (final media: SC-ARG, −HIS, −LYS, −LEU, +canavanine, +thialysine, +geneticin, +hygromycin, and +nourseothricin) at 20° in 96-wells plates and grown to saturation. Cultures were spotted on solid agar-plates in 384-format using a Biomek FX robot [Beckman Coulter (UK) Limited, High Wycombe, UK], and growth assays carried out as in Addinall *et al.* (2011), except we used concentrated cultures, without diluting in water, for *lyp1Δ* (control), *pol1-4*, *pol2-12*, and *cdc2-2* experiments. Plates were imaged automatically, usually every 4 hr. For each genotype, we performed four independent genetic crosses and analyzed two growth curves from each cross (eight growth curves in total). In some cases, however, we examined more than eight cultures (*i.e.*, up to 144 for *his3Δ*, repeated across several plates) and for others less than eight (*i.e.*, down to four because of to technical errors). The specific number of growth curves analyzed for each double mutant can be found in File S1.

### QFA fitness estimates

Cell-density estimates are generated from time-course photographs using Colonyzer2 [(Lawless *et al.* 2010), http://research.ncl.ac.uk/colonyzer/]. Strain fitness was quantified by first fitting the generalized logistic model to growth curves:

$$\frac{dx}{dt}=rx\left(1-{\left(\frac{x}{K}\right)}^{\nu }\right)$$

where *t* is time since inoculation, *x* is cell density, *r* is a growth rate parameter, *K* is carrying capacity, and *ν* is a parameter controlling symmetry of the growth curve. This differential equation has an analytical solution:

$$x\left(t\right)=\frac{K}{{\left(1+\left({\left(\frac{K}{x_{0}}\right)}^{\nu }-1\right)e^{-r\nu t}\right)}^{\frac{1}{\nu }}}$$

where *x*_0_ is the cell density at inoculation (assumed constant for all spots on all plates). We derive expressions for maximum doubling rate (MDR) and maximum doubling potential (MDP) for this generalized logistic model, equivalent to those presented by Addinall *et al.* (2011) for the logistic model [see qfa R package (qfa_0.0-39, http://qfa.r-forge.r-project.org/) for details]:

$$MDR=\frac{r\nu }{\log\left(1-\frac{2^{\nu }-1}{2^{\nu }{\frac{x_{0}}{K}}^{\nu }-1}\right)}\;\;\;\;\;MDP=\frac{\log\frac{K}{x_{0}}}{\log2}$$

Following Addinall *et al.* (2011), we define fitness as the product of MDR and MDP.

### QFA genetic interaction strength estimates

To estimate the strength and significance of genetic interaction with a query background (*xyz*), we compared the fitness of each strain in our collection (*yfgΔ-d*) in that background (*xyz yfgΔ*) with the equivalent fitness in a control background (*lyp1Δ yfgΔ-d)* under the same temperature and on the same growth media. We first fit a regression, equivalent to Fisher’s multiplicative model of genetic independence [see (Addinall *et al.* 2011) for details], to mean fitnesses across the collection, using the lm function in R:

$$\left[\tilde{xyz\;yfg\Delta }\right]=m\left[\tilde{lyp1\Delta \;yfg\Delta }\right]$$

where square brackets denote fitness and tildes denote average (mean) values for each strain. Assuming that most strains in the collection do not interact with the query background, estimating the slope *m* allows us to predict the fitnesses of individual strains in the query background, given fitness observed in the control background:

$${\left[xyz\;yfg\Delta \right]}_{pred.}=m{\left[lyp1\Delta \;yfg\Delta \right]}_{obs.}$$

If the mean of predicted fitnesses for a strain is significantly different from the mean of observed fitnesses for that strain that suggests that the strain interacts with the query background. We carried out unpaired, two-tailed tests of the null hypothesis that ${\left[xyz\;yfg\Delta \right]}_{pred.}$ is equal to ${\left[xyz\;yfg\Delta \right]}_{obs.}$ using the t.test function in R. We corrected for multiple testing by applying a false discovery rate correction to the vector of *P*-values for the strain collection using the p.adjust function in R. This gave us a vector of corrected q-values. We defined interactions with q < 0.05 as significant. We defined genetic interaction strength as the difference between the mean of the observed fitnesses and the mean of the predicted fitnesses.

$$GIS_{yfg\Delta }={\left[\tilde{xyz\;yfg\Delta }\right]}_{pred.}-{\left[\tilde{xyz\;yfg\Delta }\right]}_{obs.}$$

### Stripping genes from QFA analysis

Genes within 20 kb of query mutations were stripped from analysis because of genetic linkage (Tong and Boone 2006). In addition, strains that did not complete SGA, such as genes required for mating and the biosynthesis of histidine, lysine, arginine, and leucine, were excluded as well as genes that failed the transition between SGA and QFA (probably due to prolonged storage on plates at 4°). Lists of genes stripped in each screen are available in the File S2 and in the GISReports folder on the following Web site: https://github.com/lwlss/profilyzer_Dubarry2015.

### Gene ontology (GO) terms analysis

A list of enriched biological processes and biological components was downloaded from the Gene Ontology enRIchement anaLysis and visuaLizAtion tool (GOrilla: http://cbl-gorilla.cs.technion.ac.il/) using a p-value threshold of 10^−3^ (Eden *et al.* 2009). Significant negative or positive genetic interactions for each screen were compared with all genes screened as a background gene set to identify enriched GO terms. The lists were filtered to include only terms that annotated between two and 250 genes. Final lists were ranked by “enrichment” score (Table S3, Table S4, and Table S5) and filtered to include only the smallest terms of any branch ranked at the eighth level, or below, of the GO hierarchy (see Figure S3). p-value is not corrected for multiple testing. The q-value is adjusted to control for the false discovery rate (< 0.05) (Klipper-Aurbach *et al.* 1995). The dates when enrichments were assessed are indicated in each table.

### Dynamic interactive X-Y (DIXY) plots

DIXY is an online data visualization tool for browsing QFA fitness plots, which show evidence for genetic interaction by comparing fitnesses of strains in a query screen with fitnesses in a control screen (Addinall *et al.* 2011). QFA screen data presented in this paper along with data from (Andrew *et al.* 2013) can be visualized here: http://bsu-srv.ncl.ac.uk/dixy-pol/viz/. Note that the DAmP alleles are missing from the data obtained in Andrew *et al.* (2013). The DIXY Web site includes documentation to help users interact with plots. DIXY uses the Python web-framework Django (https://www.djangoproject.com/) to serve dynamic web pages powered by the javascript libraries D3 (http://d3js.org/) and jQuery (http://jquery.com/). The tool requires genetic interaction strength (*GIS.txt) files as input, as generated by the R package for QFA: http://qfa.r-forge.r-project.org/.

### Profilyzer

Profilyzer is an online tool for visualizing and interrogating the results of multiple QFA screens at once. QFA screen data presented in this paper, along with data from Andrew *et al.* (2013), can be visualized here: http://research.ncl.ac.uk/qfa/Dubarry2015/. Note that the DAmP alleles are missing from the data obtained in Andrew *et al.* (2013). The Profilyzer Web site includes documentation to help users interact with plots. Profilyzer is built using the Shiny framework (http://shiny.rstudio.com/) as a wrapper around a set of custom-built R functions. Source code and data underlying this instance can be found on GitHub: https://github.com/lwlss/profilyzer_Dubarry2015.

### Data availability

All strains and materials are available upon request. File S1 and File S2 contain the raw data from the screens performed in this study. These information are also available through GitHub: https://github.com/lwlss/profilyzer_Dubarry2015 with description of the fitness estimates (generated by the qfa R package http://qfa.r-forge.r-project.org/) and description of the Profilyzer tool. Source code for the DIXY visualization tool can be found at https://github.com/Bioinformatics-Support-Unit/dixy. Table S1 contains the list of strains used in the study. Table S2 summarizes the positive and negative interactions identified (q < 0.005). Table S3, Table S4 and Table S5 show enriched gene ontology terms for the DNA polymerase mutants. Table S6 provides a description of all the negative interactions affecting the DNA polymerase mutants.

## Results

We performed QFA to uncover genetic interactions affecting the fitness of strains containing hypomorphic, ts alleles affecting each of the catalytic subunits of the three major replicative polymerases. We examined *pol1-4* affecting Pol α function, *pol2-12* affecting Pol ε function (Budd and Campbell 1993), and *cdc2-2* affecting Pol δ function (Hartwell *et al.* 1973) (Figure S1).

First, at permissive temperature, we crossed polymerase-defective strains to a genome-wide loss of function library using the SGA method (Tong *et al.* 2001). The library contained nonessential gene disruptions (*yfgΔ*, replaced with the KanMX marker) and DAmP alleles affecting essential genes [*yfg-d*, DAmP, which can partially reduce mRNA stability by inserting the *KanMX marker* into the 3′UTR of essential genes (Breslow *et al.* 2008)]. We indicate the combined library as *yfgΔ-d*. Fitness of strains was deduced from growth curves of cultures spotted on solid agar. Individual cultures were monitored by time course photography and curves fitted to the data allowing us to estimate MDR and MDP for each (see Figure S2 for a small set of examples). Generally, for each genotype, eight curves were analyzed. Fitness was defined as the product of the MDR and MDP, as before (Addinall *et al.* 2011). We performed QFA at temperatures that reduced fitness of strains with ts alleles to approximately 40–60% of wild-type fitness. In total, approximately 230,000 growth curves were generated (File S1).

### DNA polymerase screens

**Figure 1:**
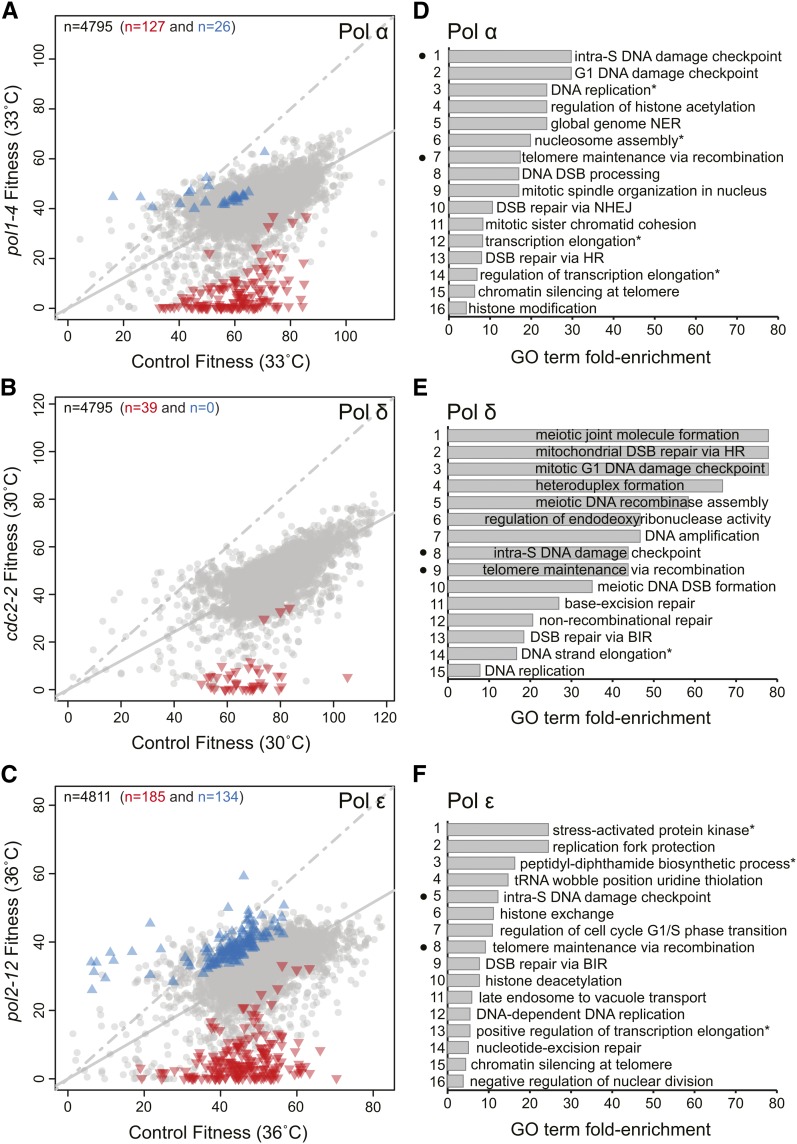
Genetic interactions that affect DNA polymerase functions. (A) Fitnesses of yeast strains with *yfgΔ-d* mutations in *pol1-4* or *lyp1Δ* genetic backgrounds and cultured at 33°. Each symbol shows the effect of a single *yfgΔ-d* in the two contexts, and the total number of *yfgΔ-d* genes shown is indicated by the number in top left corner of the plot. The solid gray line is a linear regression through all points, and the dashed line is the line of equal fitness (if there were no fitness cost caused by *pol1-4* at 33°). Statistically significant positive genetic interactions with *pol1-4* are represented by blue triangles, negative genetic interactions by red inverted triangles, and the remaining observations by gray dots. The numbers of significant interactions are indicated by the colored numbers across the top of the plot. (B) Same as in (A) but for DNA polymerase δ (Pol δ) mutation (*cdc2-2*) at 30°. (C) Same as in (A) but for the DNA polymerase ε (Pol ε) mutation (*pol2-12*) at 36°. (D) A ranked list of enriched biological process Gene Ontology (GO) terms across the negative genetic interactions identified for *pol1-4*. The complete list of enriched terms GO terms and classifications are found in Table S3. Black dots correspond to the common terms found enriched in the DNA polymerase α-primase (Pol α), Pol δ, and Pol ε screens. The asterisk indicates that the GO term has been abbreviated from Table S3. (E) Same as in (D) but for Pol δ mutation (*cdc2-2*) at 30°. See Table S4 for the complete list of enriched terms GO terms. (F) Same as in (D) but for the Pol ε mutation (*pol2-12*) at 36°. See Table S5 for the complete list of enriched terms GO terms.

Pol α-primase initiates DNA replication of both leading and lagging strands. Figure 1A shows the effects of about 4800 library mutations combined with either a Pol α mutation (*pol1-4*; *y*-axis) or a control mutation (*lyp1Δ*, *x*-axis). It is clear that at 33°, *pol1-4 yfgΔ-d* double-mutants grew on average more poorly than *lyp1Δ yfgΔ-d* mutants. We observed 127 double-mutants that grew significantly more poorly than expected (inverted red triangles), reflecting negative genetic interactions with the Pol α mutation. 26 positive genetic interactions (blue upward triangles) also were identified (Table S2).

We next examined the results obtained with the Pol δ mutation. Pol δ primarily contributes to lagging-strand synthesis but is sometimes engaged in leading-strand synthesis (Daigaku *et al.* 2015; Flood *et al.* 2015). Interestingly we only identified 39 statistically significant negative genetic interactions with the *cdc2-2* mutation and no positive interactions (Figure 1B and Table S2).

Finally, we focused on Pol ε, which is the major leading strand DNA polymerase and in addition plays an important role in the assembly of the CMG helicase at origins of replication (Muramatsu *et al.* 2010). With *pol2-12*, we saw the largest number of genetic interactions, identifying 134 positive interactions and 185 negative interactions (Figure 1C and Table S2).

We interrogated the biological significance of the genetic interactions identified in the three screens by searching for enriched GO terms in the positive and negative interactions (Figure 1, D−F, and Figure S3). Negative genetic interactions gave enriched GO terms for Pol α, Pol δ, and Pol ε mutants, whereas positive genetic interactions only gave enriched GO terms with Pol ε mutants (Table S3, Table S4, and Table S5). The two enriched GO terms found from *pol2-12 yfgΔ-d*−positive interactions are related to cytoplasmic translation and ubiquitin-dependent protein catabolic process. The proteolytic and ubiquitin systems clearly contribute to DNA replication, but the many potential mechanisms of action remain to be fully understood (Finley *et al.* 2012).

The enriched GO terms found among the negative interactions with the different DNA polymerase mutants were enriched for numerous pathways functioning in DNA replication, DNA damage checkpoint regulation, and various DNA repair pathways (Figure 1, D−F). Interestingly, only two of 47 GO terms: the intra-S DNA damage checkpoint and telomere maintenance via recombination, were found in all three Pol α, Pol δ, and Pol ε screens. We conclude that defects in any of three DNA polymerases causes cells to rely on genes important for DNA checkpoint pathways and telomere integrity. However, the more specific nature of the most of the genetic interactions we have uncovered suggest that polymerase-specific interactions allow us to distinguish the division of labor between the three replicative DNA polymerases. If so, these data are a valuable resource to help to understand the numerous, complex, biochemical interactions combine together to result in efficient DNA replication.

### DIXY plots: an interactive data visualization tool

To better facilitate the appreciation of and interaction with these data and to compare the effects of specific genes in the context of different polymerase mutations, we have developed an online data visualization tool called DIXY (http://bsu-srv.ncl.ac.uk/dixy-pol). DIXY connects a series of fitness plots like the three shown in Figure 1. It is possible to highlight genes based on their location in one plot, their name, GO function, or presence in known complexes/pathways and determine where the chosen genes lie across the fitness plots. To help highlight different genetic interactions, the points (*yfgΔ-d* mutations) move positions as the user switches between datasets (*i.e.*, all points in Figure 1A move to different positions in Figure 1, B or C).

With DIXY, it is possible to compare the effects of mutations across datasets to identify factors that affect DNA replication in different ways. For example the *rrm3Δ* mutation, which encodes a DNA helicase that helps replisome progression in some regions of the genome (Ivessa *et al.* 2002, 2003), seems to cause particularly poor growth in the Pol ε mutants but less so in Pol α or Pol δ mutants (Figure S4). In contrast, the *sgs1Δ* mutation, which affects a different DNA helicase of the RecQ family (homologous to human BLM) that is involved in the suppression of illegitimate recombination (Myung *et al.* 2001; Watt *et al.* 1996), shows the opposite phenotype. Both Rrm3 and Sgs1 limit genome instability but this pattern shows that clear differences in the roles played by these helicases during regulation of DNA replication. Such differences in genetic interactions are informative about DNA replication processes and can be explored using DIXY.

### Specificity of genetic interactions affecting DNA replication

**Figure 2:**
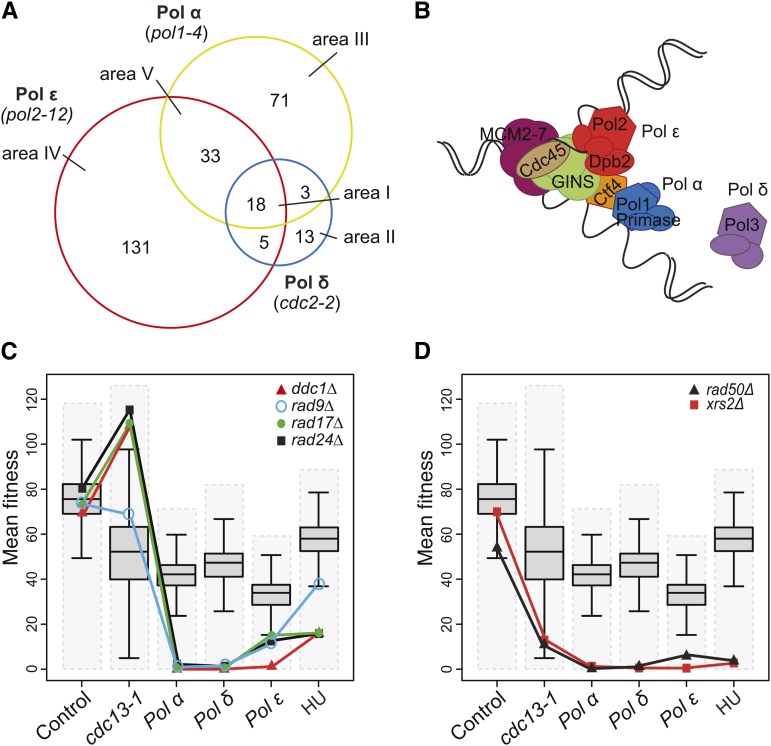
Specificity of genetic interactions. (A) Venn diagram showing overlaps between negative genetic interactions that affect cells with a DNA polymerase α-primase (Pol α) mutation (*pol1-4*), DNA polymerase δ (Pol δ) mutation (*cdc2-2*), or DNA polymerase ε (Pol ε) mutation (*pol2-12*) (Table 1 and Table S6). (B) A simplified view of the replication fork based on Sengupta *et al.* (2013). Pol ε binds to origins via Dpb2-GINS interaction. Pol ε remains associated with the CMG helicase (Cdc45-MCM2-7-GINS) during the leading-strand synthesis. Pol α-primase is coupled to the CMG helicase via Ctf4 and initiates the DNA synthesis on both strands. Pol α is replaced by Pol δ on the lagging-strand to extend the nascent strand. Note: for simplicity, numerous essential replication factors have been omitted (*i.e.*, PCNA, RFC, Dpb11 etc.) (C) Fitness profiles of gene deletions affecting the DNA damage checkpoint (*ddc1Δ*, *rad9Δ*, *rad17Δ*, and *rad24Δ*) in combination with *lyp1Δ* (control), *cdc13‐1*, *pol1-4*, *pol2-12*, and *cdc2-2* mutations and in presence of 100 mM hydroxyurea (HU). Dashed gray boxes represent the fitness range of the double-mutants in each screen. Box plots show 50% range, the whiskers represent 1.5-fold the 50% range from the box, and the horizontal black line is the median fitness. (D) Fitness profiles of gene deletions affecting the Mre11 complex (*rad50Δ* and *xrs2Δ*) as in C).

To explore the differences between the genetic networks affecting the three DNA polymerase mutants, we analyzed the distribution of specific negative genetic interactions over the three screens (Figure 2A). The interactions can be considered in the context of a current view of the eukaryotic DNA replication fork (Figure 2B). Only 18 gene deletions/DAmP mutations reduced fitness of all three DNA polymerase mutants (area I, Figure 2A, Table 1, Table S6). This small number is consistent with the fact that only two GO terms, the intra S DNA damage checkpoint and telomere maintenance via recombination, were found in all three screens (Figure 1). Much larger numbers of genes were found in other areas of the plot *e.g.*, 131 gene deletions specifically interacted with the Pol ε mutation (area IV, Figure 2A). Thus, these data reinforce the view that there are many more genetic interactions that are specific to single polymerases than interactions that affect fitness of strains defective in all three polymerases.

**Table 1:** Enhancers affecting defective DNA polymerase strains (q = 0.05)

Defective DNA Polymerase(s)	Genes
Pol α, Pol δ, and Pol ε	*BRE1 CAF40 DDC1 HTZ1 IPP1 LEA1 NCB2 POL32 RAD9 RAD17 RAD24 RAD50 RAD52 RAD57 SHR3 SMI1 XRS2 YLR339C*
Pol ε and Pol δ	*INP52 LGE1 SAD1*
Pol α and Pol δ	*BMH1 NMD2 SPE1 TEN1 YDR269C*
Pol α and Pol ε	*ASF1 CSM3 CTF18 CTF8 GIM4 HDA3 HPC2 IES5 LSM7 MRC1 MTC1 MTC4 NHP10 NST1 PAT1 PPH21 RRD2 RTF1 SOK2 SPN1 SRS2 SWC5 SWD1 SWD3 TCO89 TIF5 TIM50 UBA4 VMS1 VPS63 YGL042C YNL235C YPL205C*
Pol α	*AIM29 ALT1 APQ12 ATG3 BDF2 BUD21 BUD28 CHD1 CIK1 CLA4 CTF4 DOT1 EAP1 ELC1 ELP6 FRS2 GPB2 HIR3 IMP2’ LSM6 MF(ALPHA)1 MTC6 MUP1 NHP2 PBY1 PET20 PSY1 PUS7 REI1 RIF1 RPB7 RPC11 RPL14A RPL31B RPS0B RPS1B RPS20 RPS4A RPS9B RRD1 RSC8 RTT10 SGS1 SIS2 SKO1 SOK1 SPT2 STI1 SUI2 TIM10 TPK3 TPT1 TRM11 UPF3 YBR099C YBR100W YJR087W YKE2 YKL069W YKL075C YLR374C YMD8 YMR166C YMR245W YNL171C YOL134C YPD1 YPR050C YPR153W YPT6*::*:MRC1*
Pol δ	*CFD1 GPI15 INO2 PKR1 PMR1 PRE8 PRI1 RAD51 RAD54 RAD55 RFC5 RMI1 YNL011C*
Pol ε	*AIM4 ARC18 ARP4 ARP6 ATG21 ATO2 AZF1 BEM1 BET3 BFA1 BPH1 BRE2 BUB2 BUD27 CAT5 CDC21 CDC33 CHK1 CHS5 CKB1 CKB2 CLB2 CLB5 DCC1 DCR2 DEG1 DFG16 DIA2 DPB11 DPB2 DPB3 DPH2 DPH5 DPH6 DST1 ELF1 ELP3 EPS1 ERG3 ESBP6 ETP1 GCS1 GET3 GIM3 GPB1 GYP1 HCR1 HDA1 HIR2 HOS2 ILM1 IPK1 JJJ3 LAP2 LSM1 LSP1 LTE1 MID1 MIT1 MMS22 MRL1 MRN1 NGL2 NOP4 NPR3 NUP133 NUP188 PCP1 PER1 PET130 PKP1 PRE9 PSF1 PSF2 RIM101 RIM13 RIM21 RIM8 RIM9 RRM3 RRN9 RXT3 SAC3 SAF1 SAP190 SET3 SGF29 SHE1 SHE4 SIF2 SIR1 SIW14 SLT2 SNT1 SNX3 SOH1 SRB2 SRN2 SSO2 SWF1 SWR1 TRR2 TRS20 TRS85 TUM1 UME6 URM1 VAC8 VAM10 VHS3 VID22 VIP1 VPS13 VPS17 VPS21 VPS29 VPS30 VPS5 VPS55 VPS60 VPS71 VPS8 XRN1 YDR090C YGL046W YGR122W YGR237C YJL169W YOL050C YSC83 YSP1*

Pol α, DNA polymerase α-primase; Pol δ, DNA polymerase δ; Pol ε, DNA polymerase ε.

### Profilyzer: a tool for assessing genetic interactions in different contexts

To better understand the relevance of the genetic interactions in the three different DNA polymerase mutants, we created Profilyzer, an interactive, fitness-profiling web-tool (http://research.ncl.ac.uk/qfa/Dubarry2015/). This allows us to view the fitness profile of specific mutations across the three DNA polymerase screens and in other contexts. We also assessed the effects mutations in the context of *cdc13-1*, causing telomere defects, since “telomere maintenance via recombination” was one of only two GO terms enriched in all three polymerase screens and in the presence of the S-phase poison hydroxyurea (HU) [Figure S5, A−B, (Andrew *et al.* 2013)]. Profilyzer can be used to see fitness profiles of individual genes or groups of genes and to identify genes with similar profiles.

We first assessed the profiles of the 18 gene deletions that negatively affect all three polymerase mutants (area I, Figure 2A, Table 1). These mutations affect proteins that are important for sensing and responding to many replication defects and/or contribute to the stability or function of the DNA replication fork (Table S6). For example, DNA damage checkpoint mutations, including *rad9Δ*, *rad17Δ*, *ddc1Δ*, and *rad24Δ* (Table 1), which were all previously reported to reduce fitness of DNA polymerase mutants (Weinert 1992), were found in this group. In Figure 2C, we observe that strains with checkpoint mutations (*rad9Δ*, *rad17Δ*, *ddc1Δ*, and *rad24Δ*) are close to average fitness in control conditions, very fit when combined with *cdc13-1*, but very unfit when combined with all polymerase mutations or grown on HU. In contrast, other genes found in area I, like *RAD50* and *XRS2* (affecting two of three components of the Mre11 complex), showed a different profile. *rad50Δ and xrs2Δ* interacted negatively with all three polymerases and HU and also interacted negatively with *cdc13-1* (Figure 2D). Thus, Profilyzer allows us to see that although all six genes affect fitness of DNA polymerase mutants similarly, *rad9Δ*, *rad17Δ*, *ddc1Δ*,and *rad24Δ* can be distinguished from *rad50Δ* and *xrs2Δ* by their effects in *cdc13-1* strains. Such comparisons will be useful for helping to clarify the roles played by specific genes during DNA replication.

*rad50Δ* and *xrs2Δ*, along with *mre11Δ*, affect the three components of the MRX complex but *MRE11* was not in central area I of Figure 2A. To investigate the reason why, we generated profiles of all three genes (Figure S5C). Reassuringly it is clear the all three genes behave very similarly, apart from in the control genetic background. Therefore, the reason why *mre11Δ* was not classified, like *rad50Δ* and *xrs2Δ*, as being in area I, seems to be that in the control experiment *mre11Δ* strains were unusually sick, probably because of a technical error during the SGA procedure. Thus, Profilyzer complements statistical cut off−based approaches to explore genome wide genetic interactions.

Profilyzer also permits the search for genes that cluster similarly to any particular query mutation. When we used this function to identify the nearest fitness profiles to *RAD24* (Figure S5D), we found *RAD17*, *DDC1*, and *RAD9* were the top three fitness profiles like *RAD24*. Furthermore, the ranking and distance of these three genes from *RAD24* reflect the fact that *RAD24*, *RAD17*, and *DDC1*, encoding components of the checkpoint sliding clamp and its loader, share many functions that are distinct to *RAD9*, encoding a checkpoint mediator protein (Ngo *et al.* 2014). Profilyzer can be customized to explore subsets of data. For example, if we search for the closest profiles to *mre11Δ* and choose to ignore the control data then *xrs2Δ* and *rad50Δ* are among the top 12 most similar profiles to *mre11Δ*.

The other 12 genes in area I also could be classified based on their fitness in the context of the *cdc13-1* mutation. Nine gene deletions including *rad52Δ*, *bre1Δ*, and *pol32Δ*, behaved liked *rad50Δ* and *xrs2Δ* mutants and were unfit in all contexts (Figure S5E), whereas *rad57Δ*, *smi1Δ* and *lea1Δ* differed because they were comparatively fit in combination with *cdc13-1* (Figure S5F). Profilyzer therefore allows us to identify genes that are important in different contexts, in the specific examples highlighted, in polymerase mutants and telomere defective mutants.

### Genes that affect specific polymerase functions

#### Regulators that work in concert with Pol *δ* (area II):

We next wanted to understand those genes that seemed to affect cells with defects in specific DNA polymerases. We first examined the Pol δ−specific interactors (13 genes, area II, Figure 2A and Table 1). We found that *rad51Δ*, *rad54Δ*, and *rad55Δ*, from the *RAD52* epistasis group involved in HR repair, were in this group (Figure S6A). Other, similarly functioning HR genes *pol32Δ*, *rad50Δ*, *rad52Δ*, and *rad57Δ* were among those genes that affected all three polymerases (area I, discussed previously in *Profilyzer: a tool for assessing genetic interactions in different contexts*). The fitness profiles of *rad51Δ*, *rad54Δ*, and *rad55Δ* are somewhat difficult to distinguish from *pol32Δ*, *rad50Δ*, *rad52Δ*, and *rad57Δ* (Figure S6A *vs.* Figure S5, E−F). The major difference is that *rad51Δ*, *rad54Δ*, and *rad55Δ* are slightly fitter in Pol α and Pol ε mutants. Clearly the boundaries between the areas of the Venn diagram in Figure 2A are indistinct and areas I and II contain genes with similar roles. Other genes in area II of the Venn diagram seemed to affect the fitness of all three polymerase mutants somewhat similarly to those in area I (*i.e.*, *rfc5-d*, *rmi1Δ*, and *pkr1Δ*). In summary, analysis using Profilyzer shows that most gene deletion/DAmP mutations found to interact with Pol δ alone (area II) also interact with Pol α or Pol ε and therefore, by this criterion, are general regulators of DNA replication.

Among the Pol δ−specific interactors in area II was *pri1-d*, affecting an essential DNA Pol α-primase subunit. This is curious, because we imagined that Pri1 would be most important in mutants defective in Pol α. Interestingly when we examined the effects of *pri2-d*, affecting another subunit of Pol α-primase, we saw a similar-shaped profile of genetic interactions across the screens (Figure S6B). Pri1 and Pri2 are each essential genes disrupted by DAmP alleles, and it has been estimated that less than half of the DAmP alleles affect gene function (Breslow *et al.* 2008). Inefficiency of the DAmP allele could explain why the effects of *pri2-d* are so mild in all cases. Irrespective, it seems that based on the fitness profiles, Pri1 is particularly important in Pol δ, Pol α, and *cdc13-1* mutants and less important to Pol ε mutants. This finding suggests, perhaps, that Pri1, with the help of the accessory protein Pri2, plays critical roles in the switch from Pol α to Pol δ DNA polymerase activity during lagging strand synthesis and at telomeres.

#### Regulators that work in concert with Pol *α* (area III):

From area III, we highlight two particularly relevant genes, *CTF4* and *RSC8*, that specifically interact with Pol α (area III, Figure 2A) in Figure S6C. *CTF4* encodes a core component of the replication fork and is important for coupling the CMG helicase with Pol α (see Figure 2B) (Gambus *et al.* 2009). Strong interactions of *ctf4Δ* with Pol α suggest that the most critical role for Ctf4 is tethering Pol α at the replication fork. Perhaps as expected, *ctf4Δ* mutants also were unfit when combined with the other polymerase mutations and very unfit when combined with the telomere-defective *cdc13-1* allele. The *rsc8-d* mutation causes a defect in an essential subunit of the RSC chromatin remodeling complex (Cairns *et al.* 1996), suggesting that interactions between Pol α and the RSC complex are critical for coordination of replication with chromatin structure.

#### Regulators that work in concert with Pol *ε* (area IV):

**Figure 3:**
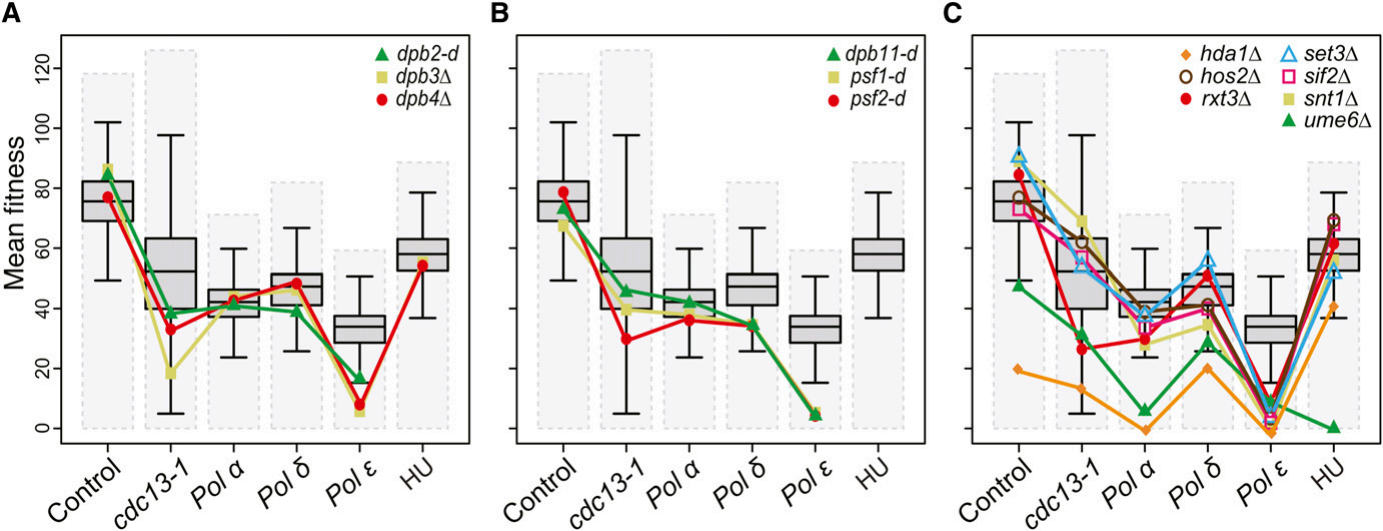
Fitness profiling of genes affecting DNA polymerase ε (Pol ε). (A) Fitness profiles of *dpb2-d*, *dpb3Δ*, *dpb4Δ* mutations as in Figure 2C. (B) Fitness profiles of components of the preloading complex: *dpb11-d* and *psf1-d*, *psf-2-d* (GINS) as in Figure 2C. (C) Fitness profiles of *hda1Δ*, *hos2Δ*, *rtx3Δ*, *set3Δ*, *sif2Δ*, *snt1Δ*, and *ume6Δ* mutations (Rpd3L-expanded complex) as in Figure 2C. Note: Note that the DAmP alleles are missing from the hydroxyurea (HU) experiments (Andrew *et al.* 2013).

The largest number of specific genetic interactors was seen with Pol ε (area IV, Figure 2A). As expected, we found other components of Pol ε: *dpb2-d* and *dpb3Δ* (*dpb4Δ* was not significant but shows a similar profile) in this area (Figure 3A). It is interesting that defects in any of Pol ε subunits affect Pol ε whereas, in contrast, defects in Pol α-primase subunits affect Pol δ function more (compare Figure 3A with Figure S6B). Pol ε has at least two major functions during DNA replication, one as the leading strand polymerase and two, as part of the pre-LC containing GINS (Sld5, Psf1, Psf2, Psf3), Sld2, and Dpb11 that forms off chromatin and is recruited to origins before replication initiation (Muramatsu *et al.* 2010). Interestingly *psf1-d*, *psf2-d*, and *dpb11-d* were also Pol ε enhancers (Figure 3B). These data are consistent with the idea that GINS-Pol ε interactions being crucial for loading of Pol ε to the replisome (in the pre-LC) and/or for maintaining Pol ε at the replication fork after replication initiation (Langston *et al.* 2014; Sengupta *et al.* 2013) (see Figure 2B).

DNA replication initiation is influenced by chromatin structure, for example, histone acetylation. Additionally, after DNA replication is completed, epigenetic chromatin marks are maintained on the replicated sister chromatids. Histone deacetylases (HDACs), particularly Rpd3, delay replication initiation (Knott *et al.* 2009; Vogelauer *et al.* 2002). Interestingly we identified genes affecting seven HDAC components as negatively interacting with *pol2-12* (Figure 3C). This was a surprise because we might expect that reduction of HDAC activity would improve the fitness of Pol ε mutants if DNA replication is easier to initiate when chromatin is more acetylated (Unnikrishnan *et al.* 2010). Therefore, this somewhat surprising result leads us to hypothesize instead that loss of HDACs, combined with defective Pol ε, leads to failure to properly replicate and maintain heterochromatic regions of the genome. Consistent with this hypothesis, in *Schizosaccharomyces pombe*, chromatin-modifying complexes travel with Pol ε to control modification of newly replicated chromatin at the fork (Li *et al.* 2011a).

#### Genes that differently interact with Pol *α* and Pol *ε vs.* Pol *δ* (area V):

**Figure 4:**
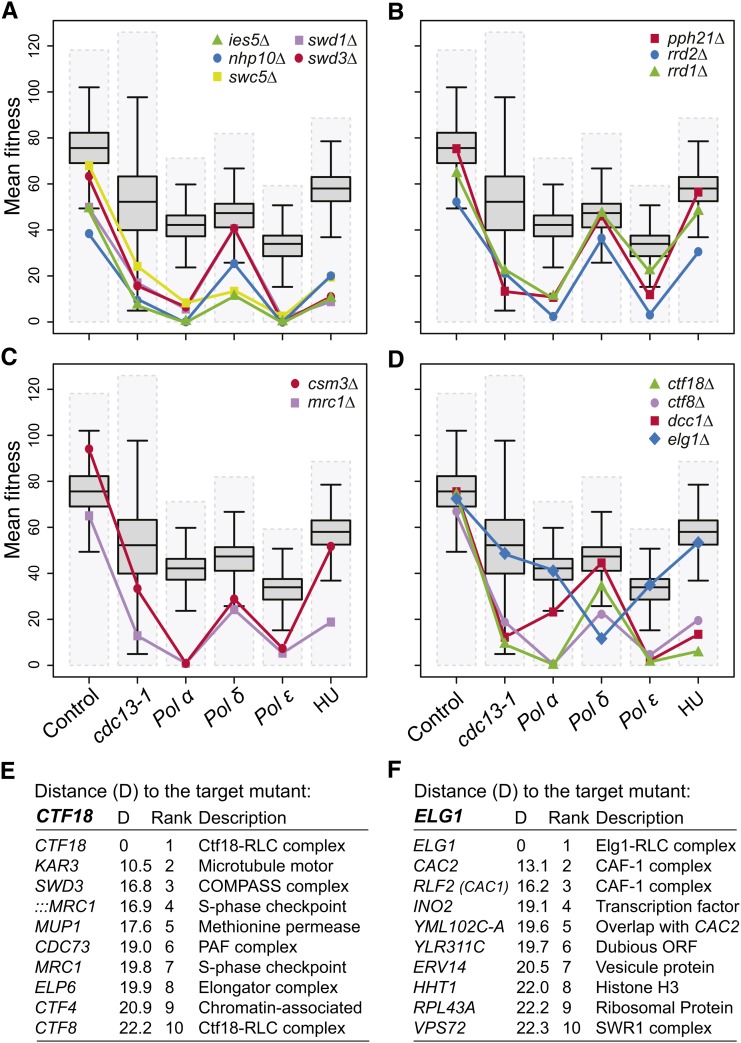
Fitness profiling genes associated with DNA polymerase α-primase (Pol α) and DNA polymerase ε (Pol ε). (A) Fitness profiles of components of the Set1-COMPASS complex: *swd1Δ* and *swd3Δ* and the INO80-type complex: *ies5Δ*, *nhp10Δ* and *swc5Δ* as in Figure 2C. (B) Fitness profiles of *pph21Δ*, *rrd1Δ*, and *rrd2Δ* mutations (Protein Phosphatase 2A complex) as in Figure 2C. (C) Fitness profiles of *csm3Δ* and *mrc1Δ* mutations (S-phase checkpoint) as in Figure 2C. (D) Fitness profiles of *ctf18Δ*, *ctf8Δ*, and *dcc1Δ* mutations (Ctf18-RLC) and *elg1Δ* mutation (Elg1-RLC) as in Figure 2C. (E) List of the top 10 gene deletions/DAmP mutations that show similar profiles to *ctf18Δ* when combined with control (*lyp1Δ*), *cdc13‐1*, *pol1-4*, *pol2-12*, and *cdc2-2* mutations and in presence of 100 mM hydroxyurea (HU). (F) As in (E) but show similar profiles to *elg1Δ* mutation.

In area V, we found genes affecting protein complexes that modify chromatin, like the INO80-type complex (*ies5Δ*, *nhp10Δ*, *swc5Δ*) and the Set1/COMPASS complex (*swd1Δ* and *swd3Δ*) (Figure 4A). Both of these complexes have been shown to promote recovery of stalled replication forks (Rizzardi *et al.* 2012; Shimada *et al.* 2008) and perhaps, therefore, this is the reason why they were in area V.

In area V we also identified two mutations (*rrd2Δ* and *pph21Δ*) affecting the PP2A complex. The *rrd1Δ* mutation affecting another subunit was in the Pol α−specific area of the Venn diagram (area III, Figure 2A) but its pattern of interactions is very similar to *rrd2Δ* and *pph21Δ* (Figure 4B). PP2A plays a critical role in the G1/S transition possibly by helping the recruitment of Cdc45, a key component of CMG complex (Chou *et al.* 2002; Lin *et al.* 1998). The recruitment of Cdc45 is necessary for CMG helicase activation and for Pol ε and Pol α recruitment to the fork (Yeeles *et al.* 2015). It is clear that *pph21Δ*, *rrd1Δ*, and *rrd2Δ* have comparatively small effects on the fitness of Pol δ mutants, suggesting that PP2A specifically affects the recruitment of the pre-LC and/or maintains stability of the core replication fork components. Pol δ is distinct to Pol α and Pol ε because it is recruited by Pol α and PCNA, rather than the CMG complex, to replication forks (Georgescu *et al.* 2015) (see Figure 2B).

Other enriched components in area V correspond to S-phase checkpoint components *csm3Δ and mrc1Δ* (Figure 4C) (Alcasabas *et al.* 2001; Bando *et al.* 2009). It is interesting that, like the *pph21Δ*, *rrd1Δ*, and *rrd2Δ* mutations discussed previously, the S-phase checkpoint genes have comparatively small effects in Pol δ mutants. Perhaps this is because these S phase checkpoint proteins and PP2A are particularly important for the stability of the replication fork (Figure 2B).

Ctf8 and Ctf18, which with Dcc1, help form the Ctf18-RFC-like complex (RLC) and load PCNA onto chromatin (Bermudez *et al.* 2003), were found in area V. Although *dcc1Δ* was not identified in area V, when we examined its pattern in different mutant backgrounds, it is very similar to *ctf8Δ* and *ctf18Δ* (Figure 4D). Rfc1 along with Rfc2, Rfc3, Rfc4, and Rfc5 forms the main RFC complex and the Ctf18-RFC complex complements RFC1 in loading PCNA during replication. Interestingly when we examined the effect of the *elg1Δ* mutation, part of an alternative RLC, we saw a very different (opposite) pattern of interactions to those seem by *ctf8Δ*, *ctf18Δ*, and *dcc1Δ* mutations. *elg1Δ* shows a strong negative genetic interaction with the Pol δ mutation but much weaker interactions with Pol α or Pol ε mutations (Figure 4D). The opposite patterns of genetic interactions seen for *elg1Δ* and *ctf18Δ* may reflect the fact that Elg1-RLC unloads PCNA whereas Ctf18-RLC loads PCNA during replication (Kubota *et al.* 2013; Yu *et al.* 2014), and PCNA-Pol δ interactions are more critical to efficient DNA binding than PCNA-Pol ε interactions, because Pol ε seems to be tethered directly by CMG (Sengupta *et al.* 2013).

### Coordination of PCNA (un)loading with histone deposition

Figure 4D, in particular, illustrates how different profiles of fitness measurements in yeast strains carrying related mutations (*elg1Δ vs. ctf18Δ*) can draw attention to different cellular functions. Therefore, we next used fitness profiling to identify the nearest fitness profiles to *ctf18Δ*. The top 10 nearest fitness profiles to *ctf18Δ*, of more than 4800 profiles created, are shown in Figure 4E and Figure S7A. Importantly, as positive controls for this approach, we identified *CTF8* as the ninth closest gene and *DCC1* was 14th closest (Figure 4E and data not shown). Other known relationships are with *CTF4*, which as we have discussed previously (in *Regulators that work in concert with Pol α, area III*), couples Pol α with CMG at the replication fork. In addition Ctf4 and Ctf18 are involved in sister chromatid cohesion (Gambus *et al.* 2009; Hanna *et al.* 2001). *MRC1* (affected by both ::*:MRC1*/*YCL060C* and *MRC1*/*YCL061C* disruptions) and *CTF18* are also important for the S-phase checkpoint pathway (Crabbe *et al.* 2010). This profiling approach found similar profiles for *SWD3* affecting the COMPASS complex, *CDC73* a member of the Paf1 complex required for gene expression, modification of histones, and telomere maintenance, *ELP6*, part of the Elongator complex, *KAR3* and *MUP1*. Further experiments will be necessary to investigate the biochemical mechanisms underlying these related patterns.

We next investigated the closest fitness profiles to *elg1Δ*, which behaved in a very different way to *ctf18Δ* (Figure 4, D and F and Figure S7B). Interestingly, the 3/4 of the closest genes to *ELG1* affect the chromatin assembly factor 1 (CAF-1) (*RLF2 (CAC1)*/*YPR018W*; *CAC2*/*YML102W* and *CAC2*/*YML102C-A*), responsible for histone deposition during DNA replication. We also found *VPS72* encoding, an Htz1p-binding component of the SWR1 complex, and *HHT1* that encodes for the histone H3. This leads us to hypothesize that Elg1 and CAF-1 orchestrate replication fork progression by coupling PCNA unloading to nucleosome assembly on lagging-strand. The HIR complex also loads histones, but this activity was defined as being independent of DNA replication in mammalian cells (Ray-Gallet *et al.* 2002). However, in yeast, it is not clear whether the role of the HIR complex is restrained to outside of S-phase. The fact that yeast mutants lacking CAF-1 subunits are viable suggests redundant pathways for histone incorporation during S-phase. On the basis of our data (Figure S7C), we suggest that the mirrored profiles of CAF-1 and HIR complexes reflect distinct activities of histone deposition during DNA replication.

## Discussion

Genome replication is perhaps the most critical task that faces any cell. Replication is a complex task, depending on numerous different proteins and nucleic acids that function together in what has been likened to a machine (Alberts 1984). A powerful combination of biochemical and genetic approaches has succeeded in identifying the core components of the replisome. The replication machinery interfaces with the cell cycle, chromatin, and transcription machinery to ensure that the processes of DNA replication are coordinated within the cell cycle and with other cellular processes. Replication machinery can falter, for example, if it encounters DNA damage, and therefore a complex series of error correction pathways help overcome difficult situations. Although the core components of eukaryotic DNA replication have been identified our understanding how these replication components are regulated by the cell cycle, or respond to error, remains far from complete. In this paper we have performed genome-wide suppressor/enhancer interaction screens to identify genes that affect the fitness of replication defective strains. We examined strains defective in the each of the three major DNA polymerases (Pol α, Pol δ, and Pol ε).

Our unbiased genome-wide experiments have identified many genetic interactions consistent with known connections between DNA replication, repair, recombination, telomere maintenance, chromatin modification, and transcription. Interestingly, most of the genetic interactions we identified were specific to individual polymerases, rather than shared across the three polymerases, which indicates that most interactions affect specific aspects of the eukaryotic DNA replication process and this pattern likely reflects the different roles of Pol α, Pol δ, and Pol ε during DNA replication. Pol ε plays a major structural role during DNA replication, as part of the Pre-LC that is so critical for initiating DNA replication and, with Pol α, a part of the core replication fork machinery. Pol δ is less structurally important (Figure 2B). We also identified interactions of Pol ε with histone deacetylases. Histone acetylation is an important aspect of chromatin replication and assembly, and histone deacetylation has been found to occur after histone deposition to allow the newly assembled chromatin to acquire higher order structure (Annunziato 2013). How or why histone deacetylation is particularly important for leading-strand synthesis will require further investigation.

Elg1 is part of an alternative RFC complex and was recently shown to be involved in the removal of PCNA from DNA (Kubota *et al.* 2013; Yu *et al.* 2014). Removal of PCNA is particularly important for lagging-strand replication because each Okazaki fragment uses a single PCNA trimer. Consistent with these recent observations, we found that the *elg1Δ* mutation particularly affects fitness of Pol δ mutants. Furthermore, *elg1Δ* caused very similar effects to *cac1Δ* and *cac2Δ* mutations in all polymerase-defective strains (Figure 4 and Figure S7). Cac1 and Cac2 encode subunits of CAF-1, involved in chromatin reassembly after DNA replication (Kaufman *et al.* 1997). Recently, it was shown that nucleosome assembly helps regulate termination of Okazaki fragment synthesis in yeast (Smith and Whitehouse 2012) and defects in histone deposition delays PCNA unloading in human cells (Mejlvang *et al.* 2014). Our results lead us to suggest that Elg1 and CAF-1 orchestrate replication fork progression by coupling PCNA unloading to nucleosome assembly. We envisage that this function is specific to CAF-1 because *hir2Δ* and *hir3Δ* mutations affecting HIRA, another nucleosome assembly complex, show an “opposite” profile to *cac1Δ* and *cac2Δ* when combined with the different DNA polymerase mutants. However, the finding that *hir2Δ* and *hir3Δ* show interactions with polymerase mutations and are sensitive to the S-phase poison HU strongly suggests that in yeast HIRA plays a role in histone deposition during the S-phase. These types of insights, built on unbiased, genome-wide genetic interaction studies, lead us to believe that our data will be a valuable resource for understanding the processes of DNA replication in the context related processes such as cell-cycle progression, transcription, histone deposition, and epigenetic inheritance.

In conclusion, we report thousands of genetic interactions that are informative about how the three major eukaryotic DNA polymerases coordinate their activities to replicate DNA.

## 
